# A Framework for User Adaptation and Profiling for Social Robotics in Rehabilitation

**DOI:** 10.3390/s20174792

**Published:** 2020-08-25

**Authors:** Alejandro Martín, José C. Pulido, José C. González, Ángel García-Olaya, Cristina Suárez

**Affiliations:** 1 Departamento de Sistemas Informáticos, Universidad Politécnica de Madrid, 28031 Madrid, Spain; 2 Departamento de Ingeniería Informática, Universidad Carlos III de Madrid, 28911 Leganés, Spain; jcpulido@inf.uc3m.es (J.C.P.); josgonza@inf.uc3m.es (J.C.G.); agolaya@inf.uc3m.es (A.G.-O.); 3 Virgen del Rocío University Hospital, 41013 Seville, Spain; cristina.suarez.exts@juntadeandalucia.es

**Keywords:** user profiling, rehabilitation, social robot, machine learning

## Abstract

Physical rehabilitation therapies for children present a challenge, and its success—the improvement of the patient’s condition—depends on many factors, such as the patient’s attitude and motivation, the correct execution of the exercises prescribed by the specialist or his progressive recovery during the therapy. With the aim to increase the benefits of these therapies, social humanoid robots with a friendly aspect represent a promising tool not only to boost the interaction with the pediatric patient, but also to assist physicians in their work. To achieve both goals, it is essential to monitor in detail the patient’s condition, trying to generate user profile models which enhance the feedback with both the system and the specialist. This paper describes how the project NAOTherapist—a robotic architecture for rehabilitation with social robots—has been upgraded in order to include a monitoring system able to generate user profile models through the interaction with the patient, performing user-adapted therapies. Furthermore, the system has been improved by integrating a machine learning algorithm which recognizes the pose adopted by the patient and by adding a clinical reports generation system based on the QUEST metric.

## 1. Introduction

Cerebral palsy is a disease affecting children, and it is notorious for causing a reduction in their movement capacity, primarily in the upper body, preventing the extremities to move as they should. This illness is characterised by its lifelong effects, hindering patients from performing essential actions such as grasping a glass. Its origin is caused by a motor disability produced by a static, non progressive lesion in the brain [1].

Although there is no cure for this disease, there exist treatments which play a fundamental role to stop it from progressing. The main option relies on physical rehabilitation therapies aiming to improve the range of movements of the extremities. In situations where surgical interventions become necessary, rehabilitation therapies are also a vital step to obtain the maximum benefit [2].

Normally, rehabilitation therapies consist essentially of different sessions where a therapist guides the patient through the execution of a series of training exercises [1]. In each of these sessions, the therapist asks the child to perform some movements, many of the times showing a movement that must be imitated. Therapies are usually comprised of many sessions, and, in the course of them, it is critical to keep the child encouraged and motivated, so the maximum benefit is obtained. Pursuing this encouragement is not easy, especially when taking into account the young age of many of the patients that have to cope with this disease. At the same time, lack of attention, boredom, or feelings of hopelessness appear often in the course of the therapy [3].

Whereas this rehabilitation process has been typically addressed by a physician, Assistive Robotics has opened an extensive range of possibilities to enhance these therapies. The friendly aspect of the robot and its ability to capture patient’s attention help to create a pleasant atmosphere. However, a proper integration of a robotic solution in rehabilitation therapies is much more than including a visually attractive element: it requires creating an interactive relationship between patient and robot [4]. To make this interaction feasible, a two-way communication becomes mandatory. On the one hand, the robot receives detailed patient’s information which is used to provide feedback to its control architecture and to adapt itself to each patient’s requirements. On the other hand, the architecture constantly sends an updated rehabilitation plan to the patient.

In the field of Assistive Robotics, NAOTherapist [5] is the name of an innovative project which aims to integrate social humanoid robots in rehabilitation therapies. NAOTherapist comprises a series of components working together to improve the development of rehabilitation therapies of the upper extremities by increasing patient’s motivation and commitment. Its architecture is composed of several components with the goal of increasing the performance in comparison with a conventional therapy [6]. At the same time, therapists are provided with tools that help them to design, execute, monitor each session and to evaluate patients based on standard clinical metrics.

**Figure 1:**
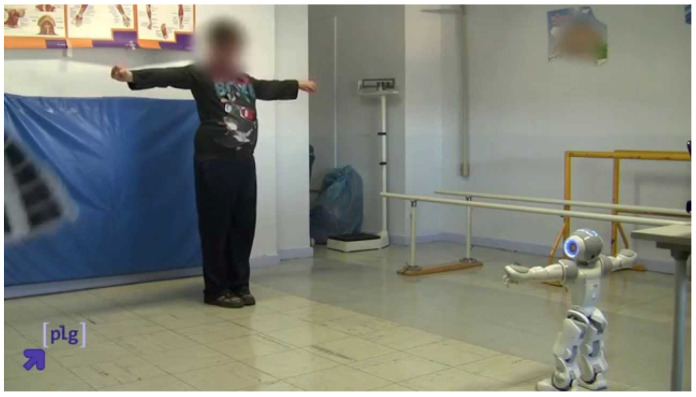
A patient with the NAO robot in a NAOTherapist session.

The main element in the NAOTherapist project is a humanoid NAO robot (https://www.ald.softbankrobotics.com/en/cool-robots/nao). It is a 58 cm high robot with 14 degrees of freedom, which allows it to move feet, legs, arms, hands and head in different directions. In addition, it also integrates different sensors such as an accelerometer or camera. The possibilities offered by this platform have made it widely used in multiple research papers and environments. In the case of the NAOTherapist project, the NAO robot allows for coaching the patient in a way that is similar to a therapist (see Figure 1).

In the course of a rehabilitation session, the robot is in charge of showing a series of predefined poses. Each of these consists of a specific position of the upper extremities where the different parts of the arm are bent in precise predefined angles. The robot first exemplifies the target posture of the arms and verbally asks the child to imitate it. Then, the system is responsible for monitoring the pose adopted by the patient by computing every angle formed in the arms and by comparing these values with the target set of angles expected. This comparison allows for defining whether the posture taken by the patient is correct or not. If it is correct, the robot proceeds to the next exercise. If the pose differs, it replicates the incorrect pose taken by the patient followed by the proper movements to adopt the correct one.

From a general point of view, the NAOTherapist architecture is oriented towards improving a patient’s condition through the use of new technologies and techniques. This includes making the patient feel more comfortable—thanks to the friendly aspect of the robot—or helping therapists with their work. A video showing the development of a NAOTherapist session is available online (https://www.youtube.com/watch?v=9n9nll28rME).

While there exists literature focused on using robots for rehabilitation therapies, there is still a big gap to be filled. Previous research [7,8,9] has already leveraged the use of Assistive Robotics to perform rehabilitation therapies. However, none of them integrate the necessary instruments to perform a complete and patient-adapted therapy together with an automatic clinical metric-based assessment of the patient’s condition.

Since each patient has specific requirements, the robotic architecture must tailor to each of them, developing a personalised therapy focused on the movements affected by the disease. This makes it necessary to generate a user profile model for each patient containing a full description of his/her condition, allowing for generating personalised therapy plans. Moreover, this user profile model must be capable of being modified in the course of the therapy, since the patient’s requirements can change. This profiling process is also performed in classical therapies, where patients are evaluated by means of clinical metrics, allowing for evaluating the improvement session by session. Another key benefit of the use of clinical metrics is that they provide a useful mechanism to share the information among specialists.

Depending on the kind of illness or the age of the patient, among many other factors, there can be different specific metrics to employ. In the context of metrics specifically designed to evaluate patients suffering from cerebral palsy, QUEST (Quality of Upper Extremity Skills Test) [10] or Melbourne Assessment [11] are the most appropriate to model each user profile. They provide a full assessment of the patient condition described in a standardised report, useful for therapists who need to share this knowledge with other specialists or with the patients. They require a therapist to fill a report with multiple fields based on the range of movement of specific joints of the patient’s body.

**Figure 2:**
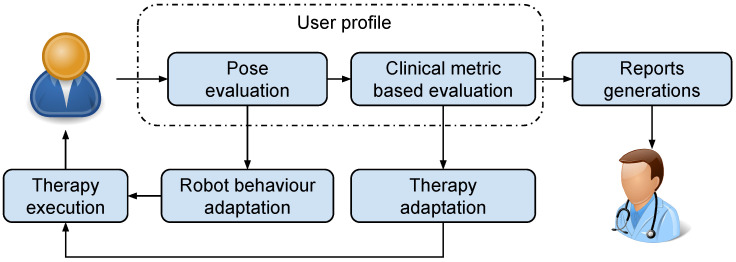
User profiling integration in NAOTherapist.

In NAOTherapist, a Kinect sensor is in charge of monitoring the movement of each part of the patient’s joints in real time. This enables to constantly compare his posture with the target posture and to determine if it is correct or not. Furthermore, the data received from this sensor can be used to analyse the range of movement of the extremities in the same way that the therapist does. Once extracting this information and evaluating it according to a standardised clinical metric, it can be used to improve the NAOTherapist architecture (see Figure 2). On the one hand, this information can be used to build a user profiling method, thus considering the particular requirements of the patient and consistently adapting the behaviour of the robot. On the other hand, it can be used as a source of information to automatically fill clinical reports. In NAOTherapist, the clinical metric used is the QUEST scale (The QUEST scale is available at: https://canchild.ca/en/shop/19-quality-of-upper-extremity-skills-test-quest), which is specifically designed for the evaluation of the upper limbs of patients with cerebral palsy. Moreover, this metric can be applied to small children, who indeed are the target population of NAOTherapist.

In a previous work [12], we briefly described the new software components that have been integrated in the NAOTherapist architecture. From then, different evaluation sessions have been performed at Hospital Virgen del Rocío, Seville (Spain), according to the time availability of physician. In this paper, we look deeper into these tools, showing how user profile models able to describe the patient’s condition are generated, how the robot behaviour is continuously adapted to the patient’s requirements, how the the clinical reports are automatically filled and how the system can learn to detect and evaluate patients using Machine Learning techniques, which allows to further extend the ability of the platform to adapt to the patient condition. This complements the previous existing component based on the use of Automated Planning, in charge of designing a whole therapy plan specifically based on each patient’s needs.

This paper is organised as follows. In Section 2, a summary of the related work is provided. Section 3 describes the whole system, explaining how the user profiles are generated and how the architecture adapts itself to the patients through a session monitoring system. In Section 4, the pose detection mechanism based on a Machine Learning algorithm is presented and evaluated. Finally, Section 5 presents some conclusions.

## 2. Related Work and Background

Robots have been proposed to help humans in a wide spectrum of tasks, as for example those related to health or elderly care. Socially Assistive Robotics (SAR) is the general term given to those robotic solutions focused on tasks such as performing medical supervision, therapies, enhancing varied skills or just trying to improve the motivation of the patient, among many others, where the interaction between patient and robot plays an important role [13]. SAR is a middle way between Assistive Robotics (AR), which tries to help the patient through physical interaction, and Socially Interactive Robotics, where the interaction does not require physical contact.

NAOTherapist is an implementation of SAR to help patients with cerebral palsy, improving the range of movement of their extremities. This architecture is part of a larger research project called Therapist [14], where different institutions are working together in a large architecture for neurorehabilitation assistance with friendly robots.

In addition to the NAOTherapist project, there is more literature in the same direction. NAO robots and a Microsoft Kinect camera have also been used with a similar approach [7]. In this research, the authors employ a Kinect sensor to monitor patient’s movement, allowing for automatically calculating two measures: Range of Motion and Dynamic Time Warping. These are, however, simple metrics which do not allow to make a comprehensive evaluation of the patient, in contrast to metrics such as QUEST which provides a score to quantify the patient’s ability to move. NAOTherapist is designed to constantly analyse the posture of the patient and to automatically generate a detailed report of a clinical metric at the end of the session. Another advantage of NAOTherapist is that its architecture is conceived to work in real time reducing, but not preventing, the presence of the therapist. There is also other work [8] focused on using humanoid robots for patient rehabilitation, but where the evaluation process is not as complete as the designed in this research for NAOTherapist, since a clinical metric is not used. “KineTron” robot [9] is also able to play the role of the therapist. However, this research only presents a system to show movements, without including tools to design the therapy or to evaluate the patient. A NAO robot and a Kinect camera have also been used with children with autism [15].

The NAOTherapist architecture pursues reaching a rich human–robot interaction, trying to build a pleasant environment with encouraging messages. For instance, the robot tries to show a friendly aspect by playing sounds or by showing coloured lights in the robot eyes. Different works have deeply studied this bidirectional communication. The ALIZ-E project [16] concentrates on these long-term social interactions, showing the importance of natural language communication, user modelling, in order to adapt the robot to the particular user characteristics, long-term memory structures or to show emotional expressions. The personalisation of this interaction has been highlighted when dealing with children [17]. According to Tapus et al., this human–robot interaction requires a robot with social skills and that shows a level of activity in line with the one exhibited by the patient [18]. A deep survey of Rossi et al. provides a wide picture of the literature related to user profiling and behavioural adaptation [19].

From a broader perspective, research has also studied and proposed solutions to enhance rehabilitation therapies with computer-aided methods but without the interaction with a robot. For instance, computer vision has remained as a useful instrument to improve rehabilitation therapies. Research on this field has implemented a serious game for rehabilitation therapies with adults with cerebral palsy in combination with a computer vision system that allows for recording every body movement [20]. One of the most important advantages of this technology is that it avoids holding physical devices, a task which some patients are not capable of doing. The use of computer vision has an extensive use in the rehabilitation therapies. Further examples include a solution to monitor a Vojta therapy with patients suffering from a neurological illness causing motor disabilities [21], an approach to improve the necessary skills to interact with a computer [22] or to use mirror feedback to enhance the results of a therapy [23].

There are also examples of other computer-aided technologies that instead of computer-vision techniques rely on other approaches. For instance, the use of biofeedback training with light, sound or colours has been studied as a means to improve cognitive rehabilitation [24]; Virtual Reality can be used to improve the treatment of primary impairments of different disorders [25]; Video Games Applied can be applied to the train the movement of the upper extremities [26]; or speech recognition tools allow for improving language skills [27]. Other researchers have also proposed architectures where a non-humanoid robot helps the patient to perform a series of movements. For instance, HipBot, a non humanoid robot with 5-DOF, helps to train different movements in the hip in order to perform rehabilitation in patients with disorders or fractures. The robot is able to perform combined movements of abduction, adduction, flexion, and extension [28]. Another example is Physiotherabot, a 3-DOF robot developed to train the lower limbs of a patient with spinal cord injury, stroke, or muscle disorder [29]. This robot performs exercises but also learns specific exercise motions.

In case of Social Robots, these have been intensively used in patients with autism, demonstrating their benefits for improving the ability to interact and socialise with other people [30]. Just like cerebral palsy, autism also requires an effort to capture the patient’s attention, where the social robot plays an essential role. For instance, robots such as KiliRo have been used to improve social interaction in patients with autism spectrum disorder [31]. A research studying the sociopsychological and physiological effects of using this robot showed a reduction in the stress levels [32]. The NAO robot has also been successfully employed in therapies to treat this illness [33]. Robots have also been studied in patients with dementia symptoms. In this case, a therapeutic companion robot called PARO showed significantly different results on the patients [34]. One of the most important benefits of using Social Robots in patients suffering from these disorders lies in their ability to promote interaction, which increases their social skills [35].

Recent research has also studied the combination of machine learning and Assistive Robotics. Particularly in the case of rehabilitation therapies [36], machine learning techniques have been used to adapt and modify the therapy plan based on the patient physiological reactions. Similarly, the use of different machine learning algorithms has been tested in order to guide rehabilitation planning [37]. With a different purpose, machine learning has been used to determine the intensity of a certain affective state [38].

Conversely, research on automated user profiling methods based on clinical metrics can be considered as limited. Instead, existing literature focuses on the use of these metrics to evaluate the patient’s condition [39], where the authors emphasise mainly on the vision system and on how to achieve an accurate virtual model of the patient posture. The Kinect sensor (In this research, the first version of the Microsoft Kinect sensor has been used) employed in NAOTherapist allows a high precision monitoring of the patient, providing an interface to easily recover and analyse the angles formed in the patient’s body. These values are used later to compare the robot and patient postures.

The combination of an automatic evaluation system based on clinical metrics with an autonomous therapies guiding system represents a gap not enough covered by current research. Nevertheless, clinical metrics have been used with non-social robotic platforms. For example, Fugl–Meyer [40], which is one of the most used clinical metrics to evaluate patients with reduced movement capacity, is used in an architecture with robots requiring physical contact [41]. Fugl–Meyer has also been used to evaluate the effects of using robots against a control group [42], but without integrating the metric in the architecture to feedback and adapt the system.

Having varied clinical metrics, it is an important decision to choose which one is the most appropriate for the objectives of NAOTherapist. The choice relies on multiple factors which have to be considered. Each metric can be focused on a specific part of the body or it can be associated with a particular disease. Generally speaking, these scales can be classified into three categories [43]: condition-specific tests, generic tests and questionnaires. The first category is the closest one to the objectives of NAOTherapist because these metrics work over a specific section of the body, as it is the case of NAOTherapist with the upper limbs. Within this category, there are two mainly used metrics: The Quality of Upper Extremity Skills Test (QUEST) [10] and Melbourne Assessment [44].

The approach of NAOTherapist makes QUEST the most suitable metric to evaluate the patient. This is due to its focus on the upper extremities, offering a wider and more comprehensive evaluation of the patient condition, analysing more movements and measuring them in greater detail, as compared to Melbourne Assessment.

### The QUEST Metric

QUEST conforms to a rigorous metric aimed at assessing the effects incurred on the patient when receiving a therapy to improve mobility skills [10]. Particularly, this metric tries to improve previous proposed metrics. One of the major advantages of QUEST lies in the fact that it can be used as a dependable and specifically designed metric to make an exhaustive assessment of the patient’s hand mobility. Furthermore, it is specially focused on evaluating the patient through a series of rehabilitation sessions. According to the authors, the metric was evaluated at McMaster University, claiming that this metric showed excellent reliability as the reference measure.

QUEST is composed of different neuro-developmental sections to evaluate if the patient is able to perform a large set of movements. These sections involve dissociated movements, grasping, weight bearing and protective extension. Regarding dissociated movements, it includes shoulder, elbow, wrist, independent (thumb and fingers) movements and also the position of the arms during grasp and release. The second section, related to grasping, analyses the different movements required to take some objects with the hands. Weight bearing is evaluated based on different positions and protective extension movements are analysed changing the centre of gravity of the patient. All these evaluation items are included into the QUEST metric based on the fact that they represent the expected range of movement developed during the first 18 months of life.

Each of these sections contains a set of check-boxes to indicate whether the patient is able or not to perform a certain movement, allowing for describing if the movement was not evaluated as well. Depending on this fact, for each check-box, a score is given: two points for those postures which were perfectly adopted and one point for those postures which could not be completed or tested. Finally, a calculation based on counting these points is performed in order to describe the patient condition in a concise way.

According to the documentation, it takes 45 min to complete a therapy aimed at filling a whole QUEST report. The child must be seated in a chair to start the therapy. During the session, the patient will be requested to adopt a series of postures and to perform different movements, trying to encourage him/her through the use of toys, demonstrations or other techniques. Each position has to be held for at least two seconds to be considered as correctly performed.

The QUEST metric has been previously used in a project where a joystick is handled by the patient [45], but here the QUEST report requires to be manually filled by the therapist. In contrast, NAOTherapist automates the process of measuring the angles in the patient’s extremities to calculate several items of the clinical metric and the generation of reports based on it. Due to the large number of elements evaluated in QUEST, and some technical limitations to capture some of them; this research is focused on calculating a subset of them: shoulder flexion, shoulder abduction, elbow flexion, and elbow extension.

## 3. Patient Profiling, Session Adaptation and Reporting in NAOTherapist

**Figure 3:**
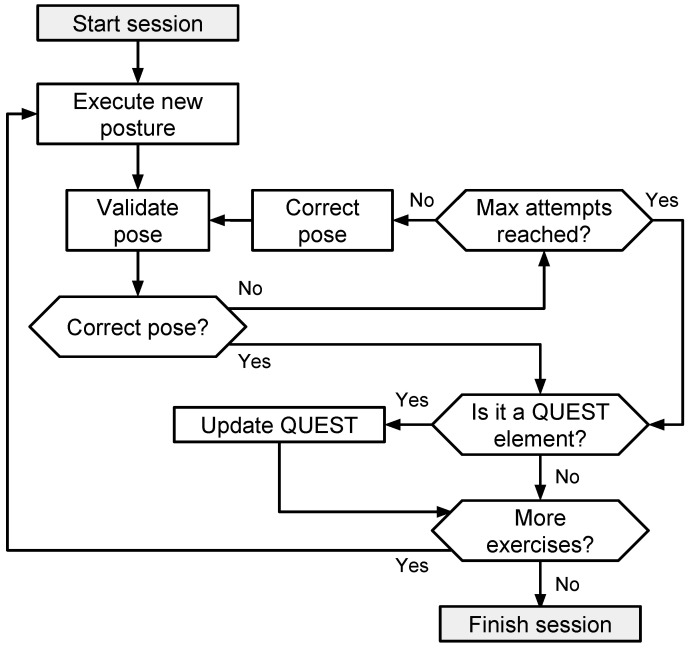
NAOTherapist session execution cycle.

A typical rehabilitation therapy for cerebral palsy involves different sessions composed of different exercises, where each session tries to improve the patient’s condition, that is to say, the movement ability, through different exercises and activities. A session within NAOTherapist (see Figure 3) follows the same approach: it is composed of a sequence of different postures that the patient has to imitate. The system evaluates if the patient’s current posture resembles the one shown by the robot and asks the patient to change the arm positions if necessary. Every time an exercise is finished, the user profile is updated according to a QUEST report, filling out different elements according to the specific movements trained in the exercise.

**Figure 4:**
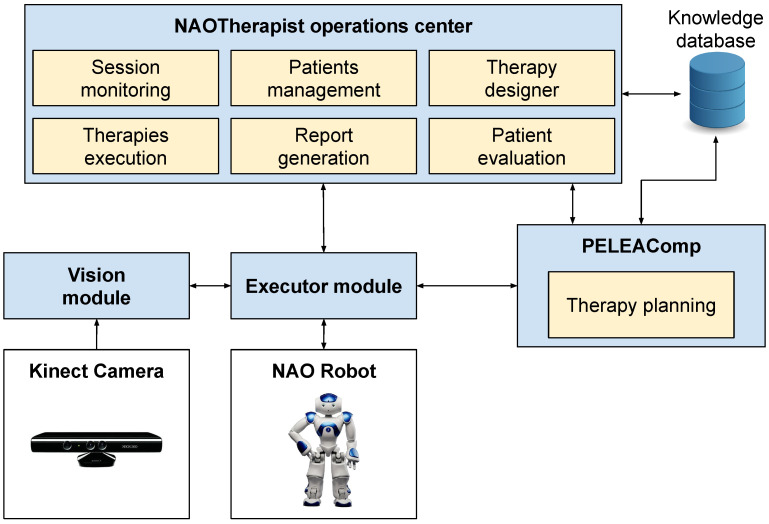
General diagram of the NAOTherapist architecture.

The NAOTherapist architecture includes a series of interconnected components which address different tasks in order to design, execute and evaluate a therapy as explained above. Figure 4 shows an overview of the NAOTherapist architecture and all the components it involves. The humanoid NAO robot, the key of the system, is the one in charge of leading the therapy. It greets the patient, encourages him during the session and performs the poses to be imitated. An artificial vision system monitors the patient position to evaluate the similarity with the required position. This system is composed of a Kinect sensor and a Vision system, which performs the comparison between the target pose and the one performed by the patient angle by angle.

During the session, both the robot and the vision system are commanded by a software which integrates Automated Planning, a subfield of Artificial Intelligence in charge of designing a plan of actions aiming to achieve some objectives [46]. The Automated Planning module operates at different levels. At the higher level, it defines the different sessions of a therapy based on a series of therapeutic objectives, a scheduling of the sessions or a number of constraints, which are provided by the therapist. At the medium level, it defines the actions that the robot has to take by using the JSHOP2 HTN [47] automated planner, which uses a hierarchical representation of the different parts of the session (warm up, middle of the session and relaxing exercises) to define every session planning. If, during the session, the architecture detects an unexpected event (i.e., the patient leaves the room or the patient is not able to complete the exercises repeatedly), a replanning mechanism is in charge of generating a new plan. At the low level, the automated planning architecture defines the movement of the joints of the robot.

By deploying a planning algorithm, the system is endowed with a mechanism that allows for dynamically adapting itself to the patient based on the information received from the monitoring module. Using this technique, it is possible to handle unexpected events, like the child sitting or leaving the session. For instance, if the system detects, through the artificial vision system, that the patient has left the area where the session is being developed, an alert is sent to the planning software, which modifies the current plan in order to include actions to warn the patient. In addition, in order to generate plans jointly coordinated with the specialist, Mixed-initiative Automated Planning has been used, a technique which is specifically designed for those tasks where human and machines work together in order to develop more effective and reliable strategies [48]. In the case of NAOTherapist, the architecture automatically elaborates the most appropriate plan of sessions and exercises by session, aiming to achieve the therapeutic objectives previously set by the therapist, but also considering the current patient’s health condition and the performance shown in previous sessions. The planning module, based on all this information, generates a plan of sessions but also defines each session, including every exercise that composes the session. The exercises are exclusively defined by the therapists, together with the therapeutic objectives they pursue.

Each exercise in the NAOTherapist architecture is composed of a sequence of poses that the patient has to imitate during a certain interval of time. In addition, each exercise is associated with a particular stage during the session (warm up, middle of the session, and relaxing exercises) and a certain level of intensity and difficulty. For each pose defined in the exercise, it is required to identify the particular set of angles. Due to the importance of the particular specification of these postures, they are defined by physicians [6]. Although there is no limitation in the number of sessions or hours that the robot can operate, the limits have been established according to the therapists’ criteria. Thus, a session and a therapy can be extended as desired. The larger the number of exercises defined, the more likely to find the appropriate set of exercises by the Automated Planning module. When designing a therapy and no suitable exercises are available in the database, the system is able to ask the therapist for new exercises complying with the patient’s requirements [49].

The mixed-initiative infrastructure requires an easy-to-use tool which enables the therapist to design and execute each session with the robot. This begins with the design of the therapy and the sessions based on the patient’s condition and expected evolution, using Automated Planning as previously presented [6].

**Figure 5:**
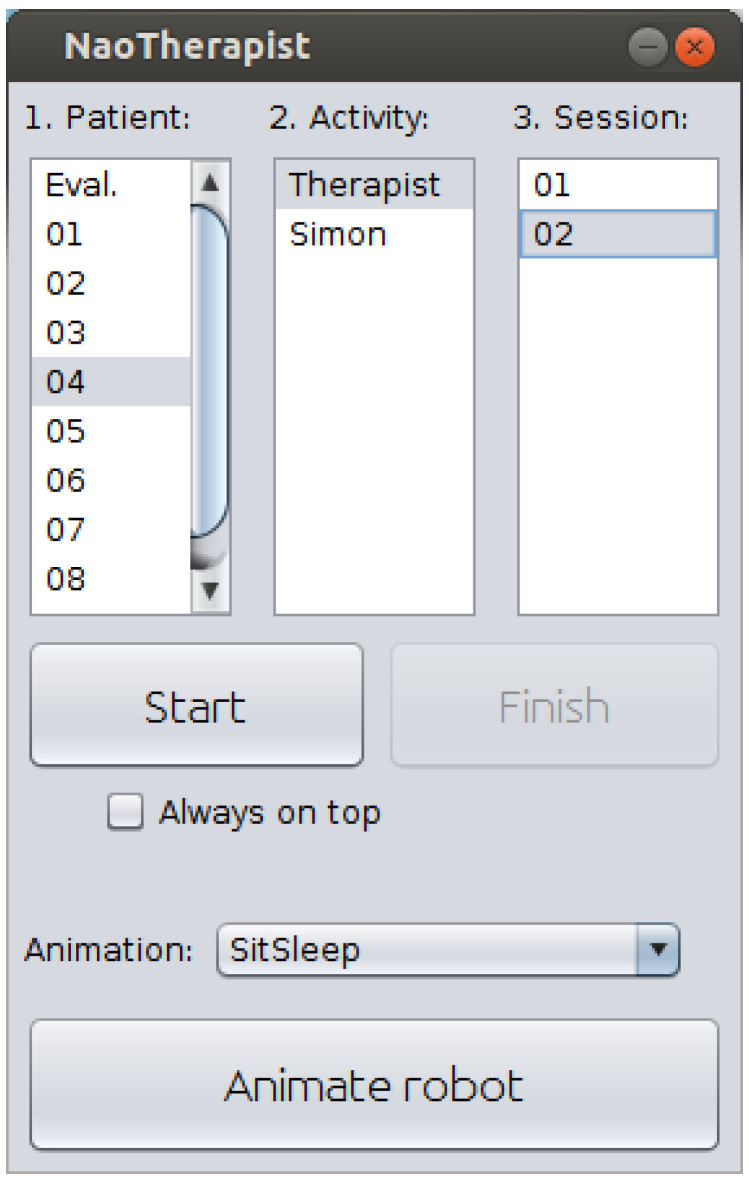
Session launch. This view allows for starting a previously defined session with a specific patient. In addition, the Simon game can also be started from this view.

Once the therapy has been defined, the therapist manually starts the execution of a particular session using the graphical interface (see Figure 5). For the time being, it is possible to start a previously defined session or the Simon game. Once a session is started, the robot will autonomously conduct the session, showing each pose, evaluating if the patient imitates it correctly, reporting feedback in case of improper performance and collecting the data needed to generate the QUEST report. The therapist can choose to stay at the room or to remotely monitor the patient’s performance through the graphical interface. At the end of each session, a QUEST report is automatically generated, allowing the therapist to modify the desired items. The tools added to NAOTherapist also allow the therapist to manage patients and therapies, providing an organised manner for accessing the saved data. The following sections cover in depth all these elements.

### 3.1. User Personalisation in Therapy Sessions

Starting a therapy involves the definition of its exercises and the use of the necessary tools to start and stop it. For this purpose, a graphical interface has been designed with the main objective of being user friendly. This program allows the robot to perform two different activities: a therapy session with its different parameters and adapted to the patient, as presented above, or a game-based session, by means of the classical Simon game [50]. This paper focuses on the first kind of therapies.

The interface allows the therapist to choose the patient, the activity to perform (as mentioned above, a regular therapy or the Simon game) and the particular session of the long-term therapy to execute. In addition, a button allows the therapist to send some simple orders and animations to the robot, as for example to greet the patient.

### 3.2. Session Monitoring and Adaptation to Patient Performance

Once a session is started, the presence or absence of the therapist during it rests in his hands (During the clinical trial performed at Hospital Virgen del Rocío in Seville, Spain, the therapist chose to stay, but the system is able to work in isolation). In any case, it is required to record data to know, at any time, how the performance was, the attitude of the patient, and any other relevant detail, such as moments in which the patient was distracted or to register if the patient leaves the area of vision. All this information allows for making a detailed user profile description. In order to provide this functionality, and also broadcasting the session to a distant place in real time, a new module, with its corresponding graphical interface, has been added to the platform.

#### 3.2.1. Monitoring Interface

**Figure 6:**
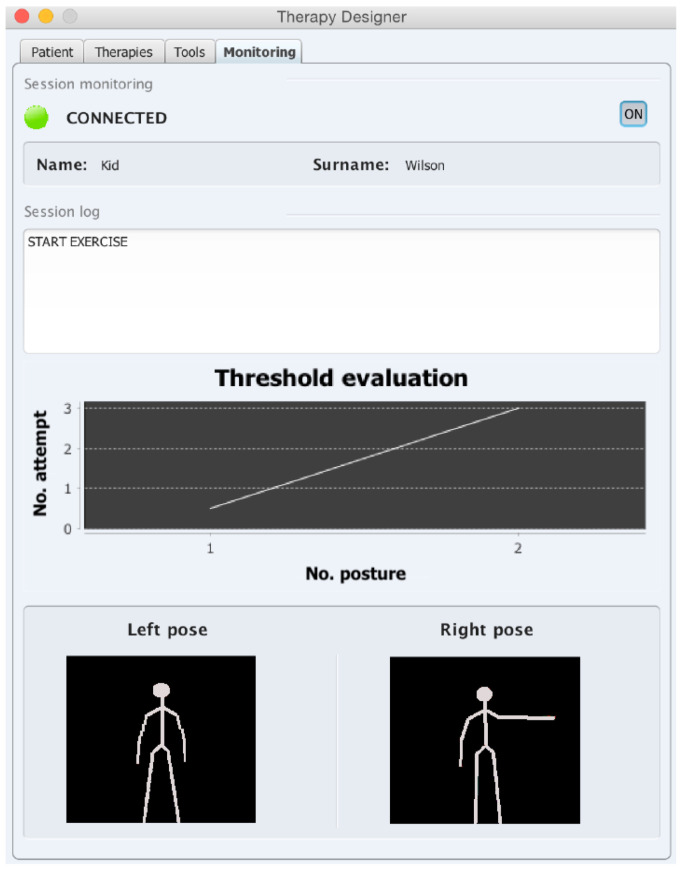
NAOTherapist monitoring interface. Real-time information is offered to the therapist, including the step that is currently being performed during the therapy session, a plot showing the number of attempts that the patient needed to complete each posture (information internally used by the architecture to adjust the evaluation threshold), and finally the last position asked of the patient.

The monitoring interface (All patients’ names have been replaced by fictitious names) (Figure 6) shows different sections providing information about the patient and the performance in the session currently being executed. This includes, from top to bottom: (a) a console with a detailed step by step information of the actions the robot is carrying out, (b) a graph showing the evolution of a threshold parameter, and (c) a picture of the pose in each limb currently asked the patient to perform. The threshold parameter θ, which is recalculated after each attempt of the patient to perform a pose, is used to adapt the architecture to the patient’s performance and to evaluate the performance over the session. In a typical session with a therapist, he/she will consider as correct certain poses even though there are differences with the requested pose. Similarly, the NAOTherapist architectures also feature flexibility when evaluating a pose. This threshold parameter defines how strict the evaluation of the correctness of a pose is; when it increases, the requirements to consider a pose as correct are relaxed, thus accepting postures that are farther away from the required ones. In contrast, θ decreases when the differences between the pose shown by the robot and the one imitated by the patient and minimal. This allows the system to stay updated on the patient’s current condition, to his limitations and to the difficulties experiment during the session. The threshold θ is present throughout the whole development of a therapy session. Its dynamic adjustment allows for considering the difficulty of every particular exercise by relaxing or by restricting the requirements to consider a pose as correct according to the patient’s performance. Moreover, it affects both limbs simultaneously, since the architecture defines sequences of poses, involving a particular position for each limb at the same time. Nevertheless, the vision system allows for evaluating both limbs individually.

The evolution of this parameter indicates the moments where the patient performs almost perfectly the exercises requested and when he fails, due to the limitations in his movements or to distractions. The aim is to relax the requirements when the patient is failing to avoid frustration. The graphic showing the evolution of this parameter is involved in the process of designing each session of the therapy, defining the exercises that the patient must perform depending on the health condition and previous sessions peer dynamically adapts it axes throughout the session for better visualisation. Regarding the two pictures shown in the bottom of this interface, they represent the position requested for each limb individually, since the internal operation of the NAOTherapist architecture works independently for each one.

#### 3.2.2. Similarity between Poses and Threshold Calculation

In a session, the patient is asked to imitate certain poses that the robot shows. The difference between the pose of the patient and the expected pose performed by the robot is calculated through the normalised Euclidean distance. Given the angles from joints $a_{i}$ where i=1...4 and $a_{i}\in$ {shoulder rotation, shoulder opening, elbow rotation, and elbow opening}. The distance $d(a^{human},a^{robot})$ is computed and normalised between 0 to 1 following Equation (1):

(1) $$d\left(a^{human},a^{robot}\right)=1-\left(\frac{1}{1+\sqrt{\sum {}_{i=1}^{4}{\left(a_{i}^{human}-a_{i}^{robot}\right)}^{2}}}\right)$$

Given $d(a^{human},a^{robot})$, the pose of the human is considered as correct if $d(a^{human},a^{robot})<=\theta$ (threshold) and is incorrect otherwise.

The adaptive threshold θ takes values from 0.28 to 0.4. Both values have been determined experimentally by the therapists. The minimum represents the strictest value to be compared with the $d(a^{human},a^{robot})$, so a more accurate imitation is needed, while the maximum is the most permissive. In every session, θ is initialised to 0.28 and is updated after evaluating the success of the patient along each pose. The system gives three attempts to perform correctly a pose, otherwise it is skipped. In this case, θ is increased a 2% (an increment fixed with the help of the therapist). The reason for this low value lies in the strong effect of the threshold when evaluating every posture. In contrast, when the patient performs correctly a pose in the first attempt, the threshold is decreased by 2%. Both cases respect the limits of θ. This explains how the system behaves being more permissive or not according to the performance and success of the patient during the session.

**Figure 7:**
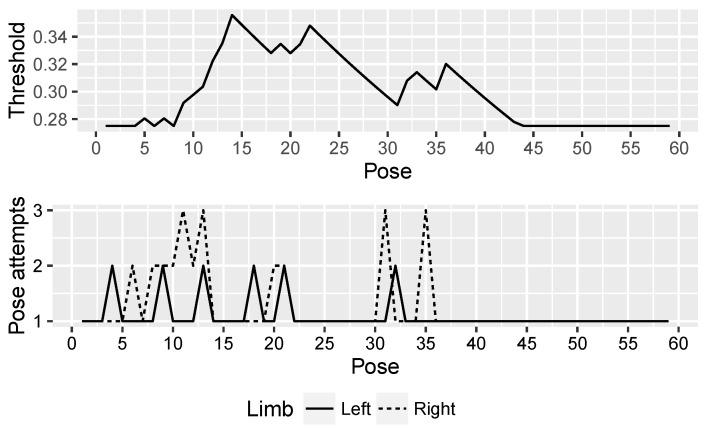
Patient P1: dynamic adaptation of NAOTherapist to the patient based on pose attempts. The top plot shows the evolution of the threshold parameter, in charge of adapting the tolerance of the system for determining a pose as correct. The bottom plot reveals the number of attempts needed to perform each pose of a therapy session. https://www.youtube.com/watch?v=9n9nll28rME.

**Figure 8:**
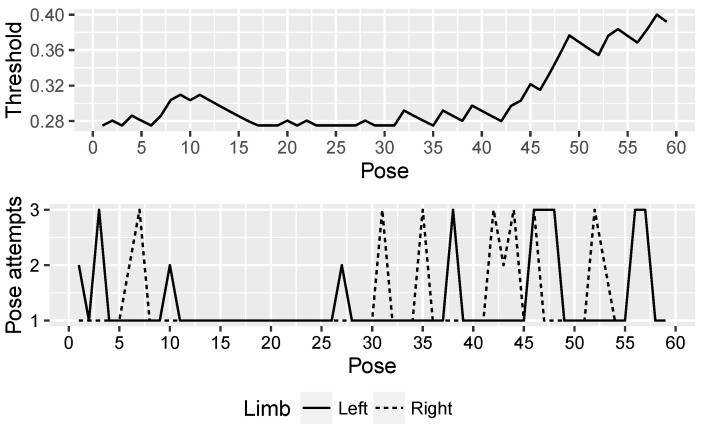
Patient P2: dynamic adaptation of NAOTherapist to the patient based on pose attempts. https://www.youtube.com/watch?v=77a20MzLVwQ.

**Figure 9:**
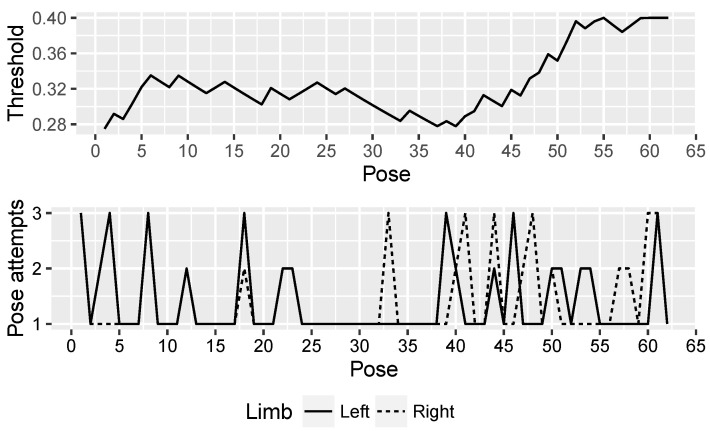
Patient P3: dynamic adaptation of NAOTherapist to the patient based on pose attempts. https://www.youtube.com/watch?v=kV-_b-sd54I.

This ability of NAOTherapist to be continuously adapted to the patient requirements offers an important improvement in its architecture. With the aim of proving the effectiveness of this new feature, three patients of the Hospital Virgen del Rocío de Sevilla attended a clinical trial where a rehabilitation session guided by the NAO robot used in NAOTherapist was conducted with each patient. Figure 7, Figure 8 and Figure 9 show the evolution of the adaptive threshold parameter (upper chart) against the attempts needed by the patient to imitate each pose executed by the robot (bottom chart). Patient P1 is a 7-year-old male and is characterised by a motor disability in the right arm. Patient P2 is a 9-year-old male with Brachial Plexus Palsy and a degree of dystonia where muscle contractions cause him twisting and unintentional movements. Patient P3 is 7-year-old male with Cerebral Palsy and physical impairments. A link to the full video of each session is provided in the caption of each figure.

The threshold starts from a low value in the three sessions and, depending on the patient’s ability to perform each exercise and the number of attempts needed to adopt the correct pose, the threshold increases or decreases. As the patient needs more than one attempt to reach a pose, the requirements for the next poses are relaxed by increasing the threshold. The patient has to hold for at least two seconds the requested pose in order to consider it as correct. If the patient is not able to adopt the pose after four seconds, the robot explains orally and demonstrates how the patient needs to modify the current position exhibited in order to reach the pose requested. This process is repeated up to three times. If the patient is not able to fulfil the position, the system discards the pose aiming to avoid a counterproductive effect, transitioning to the next pose.

As it can be seen in the three figures, the adaptive threshold progresses differently. For example, patient P1 (Figure 7) had bigger difficulties in performing the first movements, while at the end of the session executed each pose successfully on the first attempt. In contrast, patient P3 (Figure 9) experienced difficulties throughout the whole session with the exception of some poses in the middle of the session, where the threshold value decreases consistently. Finally, patient P2 (Figure 8) found the most complex part of the session in the last movements. The evolution of the adaptive threshold parameter can be used to be remotely aware of the development of the session, by merely looking at its evolution in the plot included in the monitoring screen.

### 3.3. Sessions Management and Reporting

With different patients to treat during different sessions, it is necessary to provide the therapist with tools to perform his work with adequate organisation. A new session management component allows for easily accessing data of previous and current sessions and to patients’ information. It also helps the therapist in a crucial step: evaluating the patient on the basis of a clinical metric. By recording each movement performed during the therapy execution, the system is able to make the necessary transformations to quantify the range of movements of the joints and then to automatically calculate elements of a clinical metric. This is essential to measure the patient’s evolution session by session and to generate reports following standard-based models. As previously mentioned, there are many metrics that can be used in this environment, QUEST being the most indicated for the purposes of NAOTherapist, due to its focus on the upper extremities.

#### 3.3.1. User Profiling via Quest Report Calculation

In a rehabilitation session, the therapist can manually measure different angles formed in the upper extremities to later fill the related fields in the QUEST report. This process can be made easier using the information retrieved from the Kinect sensor and its skeleton tracker module, consisting of the spatial position of each part of the body, applying different transformations to obtain the angles used in QUEST and allowing for automatically determining the related scores in the report. Once filled, this report offers a full assessment of the patient condition in a specific time, thus representing the user profile. Furthermore, since this reports follows a standardised form of describing the patient condition based on the clinical metric QUEST, it can be used to generate a similar report to the one that the therapist would write manually. This report, built as a PDF file, can later be printed or displayed on the computer screen.

**Figure 10:**
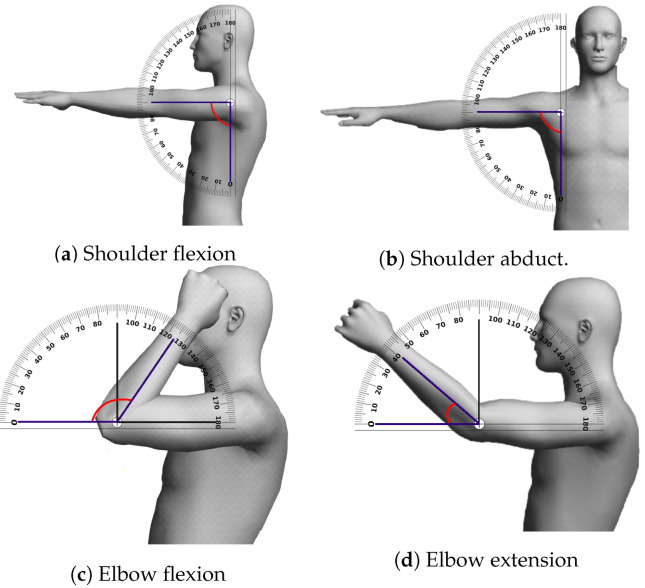
QUEST items automatically evaluated (Pictures generated with Pose Tool 3D).

The system is able to calculate four items (as shown in Figure 10) belonging to the first section of QUEST, which is focused on Dissociated Movements. The reasons for which only a subset of elements has been evaluated rely on the limitations of the Kinect sensor used, which does not allow for precisely monitoring small movements as those realised by the fingers. Our objective has been to develop an easily extensible architecture, so that, in the future, it will be possible to employ a more sophisticated sensor, allowing for filling more elements in the QUEST report.

#### 3.3.2. Evaluation of the User Profiling Method

Due to the crucial importance of the proper development of a therapy for the future of the patient, it is required to pay attention to every step in order to prevent any error that might adversely affect the patient evolution. The high degree of autonomy of NAOTherapist to perform each of these steps highlights the necessity of a deep validation, with the objective of proving if NAOTherapist succeeds in evaluating the patient’s posture, in generating a consistent user profile and checking if the reports generated are accurate.

In order to measure the accuracy of the user profiles in the form of automatically generated QUEST reports, different data were collected from three therapies performed with three different patients at the Hospital Vigen del Rocío de Sevilla. These therapies where composed of 7, 6, and 6 sessions, respectively. The results of the clinical evaluation of the system are now in development and lie out of the scope of this paper, but we have been able to use the QUEST reports created by the physician to assess the performance of our automatic QUEST report generation module. The reports generated must be similar to the ones that the therapist would manually fill. With the aim of demonstrating their validity, the system was evaluated with the three patients mentioned above, who attended therapies with the NAO robot over several weeks. At the end of the therapy, two QUEST reports were generated. The physician was asked to manually fill a QUEST report for each patient, while the system automatically delivered the second one at the end of the therapy.

From these three patients, 76 elements of QUEST were calculated. Then, the report manually generated by the therapist and the report automatically generated by the architecture were compared, calculating the number of items which were given the same score. This allowed for obtaining the accurateness of NAOTherapist when generating the QUEST report in a totally automated manner. This comparison showed that 85% of the total number of elements was given exactly the same score. In order to put this value into context, we have resorted to the survey by Novak et al. [51], where a review of the uses of machine learning for user adaptation is made. In most cases, accuracy values are under 80%, and only a few are able to obtain 85%–86% accuracy, while reaching higher values have only been achieved in very rare occasions under certain conditions. Moreover, the report was identical when we compared the automatically generated report and the one made by the therapist for two patients. These results ensure confidence in the reports automatically generated; nevertheless, the system provides the therapist with the necessary tools to make any change in the report as it deems appropriate.

The generation of these reports relies on an evaluation system which decides whether a pose imitated by the patient is correct or not. As already discussed, a Machine Learning algorithm has been implemented with the aim of adapting the robot behaviour to the current performance of the patient, calculated as a relation between the poses performed by the patient and the ones shown by the robot. Before using this model in a real environment, we have tested it using mathematical comparisons, training the model with poses formed by the angles expected, the angles achieved and a value indicating if the pose is correct according to a mathematical comparison between both limbs. Section 4 describes the Machine Learning algorithm used and proves its ability to detect correct and incorrect poses.

#### 3.3.3. Reports Management and Generation

A therapist deals with different patients having different conditions and requirements performing many sessions throughout the day. This highlights the need for a system integrating all the tools needed to manage, design, and execute therapies and also to generate clinical reports. In order to have a system which fulfils all these requirements, we have designed a graphical tool to manage all therapies and patients and their sessions, making it possible to access any session and generate and modify two reports (the QUEST report and the detailed report). To generate these reports, the JasperReports Library (http://community.jaspersoft.com/) has been used.

**Figure 11:**
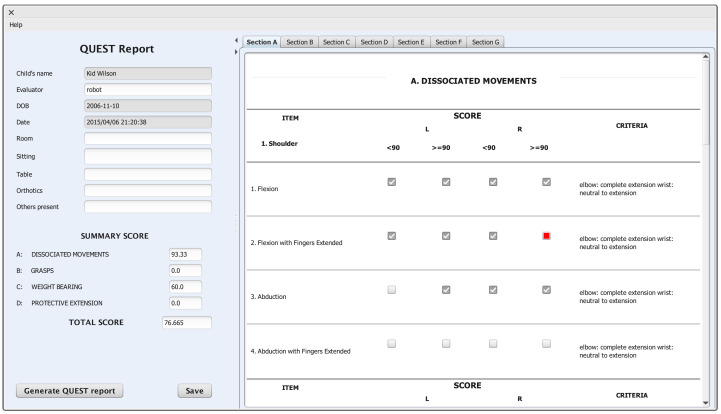
NAOTherapist sessions and reporting.

The graphical interface allows for viewing, modifying and generating the QUEST report in PDF format, as it can be seen in Figure 11. The left side of the window includes different data about the patient, the session and also the scores of the metric, which are calculated and updated automatically when any of the items are modified. The right side allows for navigating to any section of the metric and modifying the desired elements. The items automatically calculated in real time during the session by the robot will appear already filled in this view, although the clinician is allowed to modify them.

## 4. Pose Recognition Using Machine Learning

Therapies oriented to patients having difficulties with mobility try to improve their capacity of movements, increasing the range of them session after session. To this end, it is necessary to thoroughly assess the patient condition in each session and to evaluate if it improves in the next session, a task which traditionally lies on the therapist. This is critical in the development of the therapy, in order to make the necessary changes to adjust it to the new patient’s requirements. Using a robot and an artificial vision system, where the therapist presence is not mandatory, it is necessary to continuously monitor the patient and to evaluate if the poses that the patient imitates are consistent with the poses shown by the robot.

The detailed information gathered by the vision system contains every movement performed by the patient with the upper limbs, angle by angle. These data are used to compare the patient posture with the one shown by the robot (specifically designed to train and improve a specific movement), and to decide if the pose is correct or not. Depending on this comparison, the behaviour of the robot is adapted in order to show an appropriate response to the current patient performance. In order to improve the ability of the system at calculating the patient’s body position, several captures are taken and combined.

Once a patient’s body has been captured, it is necessary to evaluate if it fits the posture expected. One possible approach is to use a mathematical comparison, as shown in the previous section: if the difference (a Euclidean distance) between the requested pose and the imitated one (angle by angle) does not exceed a previously defined interval (applying a dynamic threshold as previously mentioned), the pose can be considered as correct. However, this method is too inflexible and it does not take into account the therapist’s expert knowledge, who is able to consider other relevant information to make a decision, such as particular rotations or displacement of the limbs. Furthermore, different poses would require different recognition thresholds.

### 4.1. Applying Machine Learning to Pose Evaluation

To address this problem, Machine Learning can be used to learn from the decisions made by the therapist and to generate more accurate and flexible models. The basic idea is to train the algorithm with a set of poses, including the expected angles, the ones actually achieved and the decision of the therapist about the posture correctness. Once trained, the model receives the angles and delivers an output classifying the pose as correct or incorrect.

In order to train a machine learning algorithm, it is required to gather a dataset of instances or examples, vectors composed by a set of features and a class or label. In our research, examples represent poses, composed by features which are the set of angles measured by the vision system and the target angles. The class of each of these examples defines if the pose is correct or not. By sequentially introducing these examples to the machine learning algorithm, it will learn to associate a particular set of expected and actually showed angles with a particular labelling: if the patient performed the pose correctly or not.

There are different Machine Learning algorithms for classification which can be applied to resolve this problem. However, the choice has to be made on the basis of the application domain. In a medical environment such as this, it is indeed important to obtain a self-explanatory model able to provide the grounds of a decision. Decision trees form a group of algorithms that are composed of logical rules that allow for easily extracting human knowledge, in comparison to other algorithms that are not self-explanatory (for example, support vector machines). Specifically, we have used a decision tree classifier executed with the scikit-learn library for Python [52], an implementation of the C4.5 algorithm [53], one of the most studied and used decision tree algorithms. This algorithm generates a tree-shaped model that, depending on the angles of the different joints, reaches different leaves related with specific labels, deciding whether the pose performed by the patient is correct or not. In contrast to other Machine Learning classification algorithms, the simplicity of the decision rules generated, which allow the therapist to understand how every pose is evaluated, and the high accuracy makes the C4.5 algorithm a useful instrument to identify if a pose imitated by the patient is correct or not.

In order to execute this algorithm, it is necessary to provide the minimum number of objects per leaf as a parameter. This value indicates the minimum number of cases that are needed to lie in a particular leaf to be divided into two branches. If this value takes a very high value, the size of the tree will be reduced, having only a small set of rules, which is related with an easy to read, but inaccurate model. In contrast, if this value is increased, more rules will be created raising the accuracy but also the complexity of the model. In order to obtain an equilibrium between accuracy and self-explanatory capacity, different experiments were performed with various values assigned to the parameter, showing the accuracy and the size of the tree generated for each of these values. These experiments have been performed using data extracted from 6600 poses corresponding to the sessions of the three patients involved in the clinical trial. For all the experiments, 50% of the data was used for training the model and the remaining to test it. For each experiment (i.e., for each value of the number of objects per leaf parameter), 20 executions were run and the average was calculated.

### 4.2. Results

**Figure 12:**
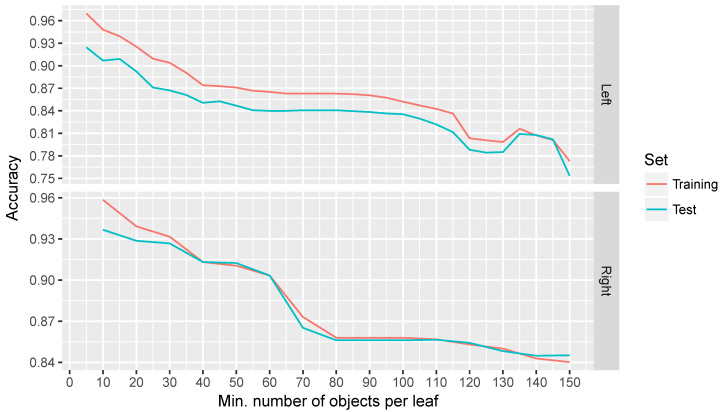
Results in terms of accuracy in the experiments performed using a decision tree classifier for both limbs.

**Figure 13:**
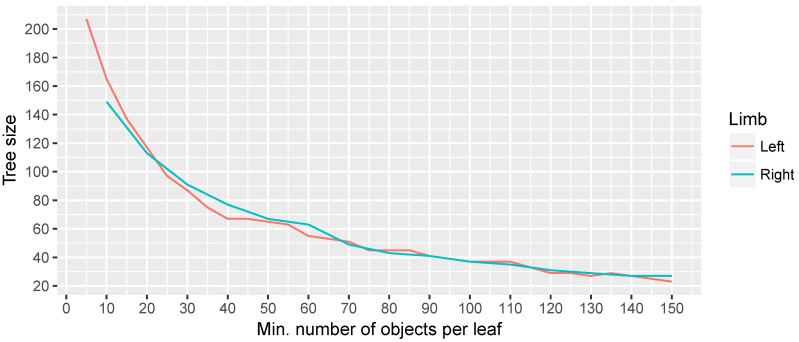
Results in terms of size of the tree in the experiments performed using a decision tree classifier for both limbs.

The results, in terms of accuracy and minimum number of objects per leaf for both limbs, are shown in Figure 12, while, in Figure 13, the evolution of the size of three is presented. In the first figure, it can be seen that there is a clear trend where the accuracy is affected by an increase in the minimum number of objects per leaf, while the size of the tree increases as expected. We consider that a minimum of 30 objects per leaf is a good compromise between the accuracy and the size of the tree. When defining a higher minimum number of objects per leaf, one can appreciate that some erratic behaviours arise.

Regarding the differences in the accuracy for both limbs, it is presumably because of lower accuracy in movements performed with the left limb by right-handed people, but needs to be studied more in detail. The accuracy, above 90%, and also its self-explanatory representation evidence that a decision tree is a good election for this problem. For this reason, this Machine Learning based method has been integrated in the NAOTherapist platform in order to better evaluate the patient posture. Nevertheless, all data retrieved from the vision system in each session are saved, including the calculation of the patient posture based on Euclidean distances as previously described in Section 3.3.2. This will allow for further improving the Machine Learning model by introducing new training examples.

### 4.3. Discussion of the Use of Machine Learning for Pose Evaluation

As described in the previous subsections, the use of Machine Learning techniques helps to evaluate the poses adopted by the patient with high accuracy while keeping a high degree of flexibility. In order to put into perspective the results obtained, we have reviewed similar literature in this field. Although there has been important progress in the symbiosis between Machine Learning and rehabilitation with social robots, there is still a long way to go. Previous research has focused on studying the use of Machine Learning techniques for recognising certain behaviours in children and adolescents with cerebral palsy with information for different accelerometers placed on different parts of the body [54]. This research showed promising results of this combination. Other research has evaluated the use of these techniques for gait classification in children with spastic diplegia [55], demonstrating that decision trees based classifiers are able to provide accurate results with high transparency, a valuable feature in this domain. In contrast to this research, the NAOTherapist architecture pursues to automate the process of filling a complete assessment metric, a task typically made by the therapist. Otten et al. [56] proposed using Machine Learning techniques to automate the assessment, in this case of the Fugl–Meyer metric. However, NAOTherapist forms a bigger architecture which covers all the steps involved in a typical rehabilitation therapy, including objectives definition or the exercises that every patient can do.

## 5. Conclusions

Rehabilitation therapies are essential to help people with movement disorders. However, in the case of cerebral palsy, the young age of the patients who have to deal with them make an effort to capture their attention and increase their motivation and interaction necessary. NAOTherapist benefits from the use of social robots to enhance the interaction with the patient and to create a more pleasant environment. A complex architecture of components offers the necessary tools to perform an entire therapy where the architecture adapts to the performance and requirements of each patient. Moreover, modelling each user condition and generating personalised user profiles enable designing specific therapy plans and generating reports based on a clinical metric. Thanks to a new dedicated graphical interface, the therapist can manually modify the report. All of these capabilities have been integrated in the NAOTherapist architecture together with a set of tools that provide a useful framework to develop the whole therapy cycle of different patients. Among these tools, there is a graphical interface which allows the therapist to create a therapy and to execute a session with the robot adapted to the patient’s requirements or an artificial vision system that continuously captures the movements of the patient and checks if they are similar to those shown by the robot. This comparison is performed by using a model obtained after training a machine learning algorithm with knowledge of an expert or by pure mathematical comparison, which allows for adapting the system over time. Currently, different studies at Hospital Virgen del Rocío of Seville conducted by therapists are assessing the benefits of the use of the NAOTherapist architecture, evaluating the improvement in the patient’s condition and psychological aspects such as the motivation and intention.

Our future work involves working on the different limitations of the architecture. Particularly, we plan to improve the vision system, including new software and hardware solutions aiming to obtain a more accurate virtual representation of the patient’s body and a more precise position of every part of the body, including the position of every finger, a feature which currently is not offered by the camera employed. Other Machine Learning algorithms will also be evaluated, since the use of a more complex vision system could require a more powerful classification model. Additionally, the number of items covered by the QUEST metric has to be increased, aiming to fulfil the whole report for every patient. New metrics such as Fugl–Meyer can also be incorporated, thus allowing the therapist to select the most appropriate one (or even a combination of them) for every patient.
